# BNP and Precision Medicine: Tip of an Iceberg∗

**DOI:** 10.1016/j.jacbts.2021.05.007

**Published:** 2021-06-28

**Authors:** David E. Lanfear, Jasmine A. Luzum

**Affiliations:** a Heart and Vascular Institute, and Center for Individualized and Genomic Medicine Research, Henry Ford Hospital, Detroit, Michigan, USA; b University of Michigan College of Pharmacy, Ann Arbor, Michigan, USA

**Keywords:** genetics, heart failure, natriuretic peptides

**Figure:**
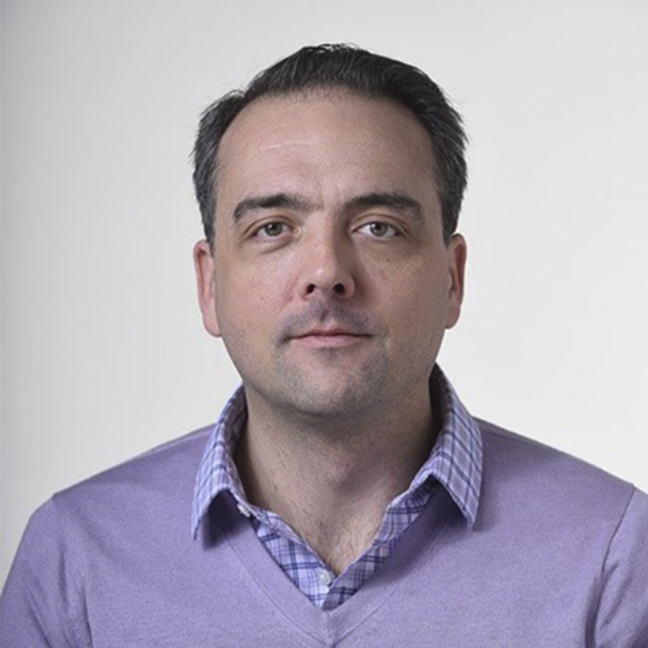

The STOP-HF (St Vincent's Screening to Prevent Heart Failure) clinical trial was a landmark, not because of gargantuan size or a novel drug, but because it was simple, practical, and yet still groundbreaking. The demonstration that our favorite heart failure (HF) biomarker, the b-type natriuretic peptide (BNP), could be used for early risk recognition and targeted intervention was a leap in the right direction. Still, the predominant view of BNP is that it is essentially a biomarker of cardiovascular (CV) risk, that is, it is only a marker and it is a bad thing. However, this is likely too narrow a view because the natriuretic peptide (NP) system has important favorable physiologic effects (not to mention a likely partial mechanism behind the benefits of sacubitril). The interesting observations in this issue of *JACC: Basic to Translational Science* from Cannone et al. (1) nicely illustrate the value of a more nuanced view of the NP system toward precision medicine.

The investigators undertook a detailed phenotypic analysis of patients in the STOP-HF trial in terms of a *NPPB* genotype. A particular genetic variant of interest (rs189389), previously associated with higher BNP production and reduced CV disease risk (2), was genotyped with the goal of better understanding the intermediate mechanisms that could be at play vis-à-vis reduced CV risk. STOP-HF is a good environment for such a study, despite its moderate size, because of its BNP-based intervention design, detailed follow-up assessments (including biomarkers and echocardiography), and relative genetic homogeneity of the study population. The authors show, as others have, that this genetic variant is associated with higher BNP levels. More importantly they elegantly extend this to demonstrate that the patients with the minor allele of rs189389 had a lower risk of developing left ventricular systolic dysfunction, lower risk of hypertension, and lower risk of adverse clinical events during follow-up. These new data fit nicely with the previous literature on this variant and more broadly with data suggesting that NP-deficient states could be an early contributor to development of CV disease and HF (3), and perhaps partially underlying the observed excess risk of CV disease in the African American community (4). On the other hand, the current results also beg for new avenues of investigation.

First is that the authors left unanswered the question of whether this genetic variant should somehow be taken into account when implementing a STOP-like program. Consistent with previous genetic data but paradoxic to traditional biomarker view, the patients with the favorable genotype were lower risk but had higher BNP levels. Should the BNP threshold for intervention (in this case referral to cardiology) be different based on genotype? And if so, what should it be? The authors may be able to tease this apart from the data they already have, but it is not apparent in the current report. More difficult to address are bigger questions about genetic screening. Being able to identify a low-risk genetic group would seem to make biomarker screening less useful in these patients and conversely more efficient in the higher-risk genotype group. Could genetic screening be used as a first layer of population screening, informing in which patients and how often BNP levels should be obtained?

Stretching beyond these direct questions takes us quickly into the realm of genomic population health. The current analysis of a single variant is simplistic but offers proof of concept, and a glimpse of a possible future. Although *NPPB* has been cited in previous genome-wide association studies (GWAS) of NP and blood pressure (5), it did not appear in the most recent and largest GWAS study of HF (6), which identified 12 additional loci associated with HF risk. Viewing these new STOP-HF findings in the larger genomic context makes it easy to imagine that combining the various genetic markers (for example, via polygenic risk scores) may be able improve risk-stratification even further. The single variant reported here seemed to help stratify risk within patients with Stage A/B HF; perhaps a more complete genomic assessment could identify patients at risk (and thus deserving of biomarker screening) even among those without established HF risk factors (pre-HF).

Finally, moving beyond disease detection to intervention, should we be developing and testing BNP-elevating strategies for HF prevention? Would this be effective, efficient, and safe? This could even be targeted to those identified to be genetically at risk for HF or NP deficiency. Answering these questions would of course be a challenge, but the biology seems to make sense, and having a congruent genetic finding is one of the best predictors of successful future therapeutic development.

In the end, this laser-focused study, in itself, cannot change our current HF paradigm. However, if we read these data with one eye toward what *could be*, observations like this one can serve to motivate and direct us toward an ever more tangible and alluring future of primordial prevention and precision interventions.

## Funding Support and Author Disclosures

Dr. Lanfear is a consultant for Amgen, Abbott, Janssen, Ortho Diagnostics, and DCRI (Novartis); has participated in clinical trials from Amgen, Bayer, and Janssen; and has a patent for a beta blocker polygenic response predictor. Dr. Luzum has reported that she has no relationships relevant to the contents of this paper to disclose.
